# Letter to the editor regarding: ‘characteristic analysis of early gastric cancer after *Helicobacter pylori* eradication: a multicenter retrospective propensity score-matched study’

**DOI:** 10.1080/07853890.2025.2550585

**Published:** 2025-08-25

**Authors:** Zhanyi Zhou, Rijuan Jin

**Affiliations:** a The First School of Clinical Medicine, Zhejiang Chinese Medical University, Hangzhou, China; b Department of Gastroenterology, Key Laboratory of Digestive Pathophysiology of Zhejiang Province, The First Affiliated Hospital of Zhejiang Chinese Medical University (Zhejiang Provincial Hospital of Traditional Chinese Medicine), Hangzhou, China

To the editor

We read with great interest the article by Liu et al. [1]. titled ‘Characteristic analysis of early gastric cancer after *Helicobacter pylori* eradication: a multicenter retrospective propensity score-matched study’. The authors comprehensively compared the clinical, endoscopic and histopathological profiles of early gastric cancer (EGC) detected after successful *H. pylori* eradication with those of *H. pylori*-infection-related EGC, and further identified several independent characteristics of the former entity. While the study provides valuable insights for endoscopists and pathologists, we would like to raise several methodological and clinical concerns that may limit the generalizability of the findings.

First, the retrospective design inherently introduces selection and information bias. Patients who underwent endoscopic submucosal dissection (ESD) during 2015–2022 were screened; however, the decision to perform ESD—and thus to enter the cohort—was not standardized across centers. This may have led to preferential inclusion of smaller, more accessible lesions, potentially underestimating the true prevalence of larger or aggressive cancers that were referred directly to surgery. A prospective registry or consecutive-endoscopy cohort would better define the real-world spectrum of post-eradication EGC.

Second, the definition of ‘successful eradication’ relied on a single negative ^13^C-urea breath test (UBT) or histology after ESD, but the timing of this test was not harmonized. Up to 30% of UBTs performed within 4 weeks of antibiotic cessation yield false-negative results [2]. Without a mandatory confirmatory test ≥8–12 weeks after eradication therapy, some ‘eradicated’ patients may have remained *H. pylori*-positive, misclassifying their cancers as post-eradication lesions and diluting between-group differences.

Third, the study excluded patients who developed EGC <1 year after eradication therapy. Although this threshold follows Saka’s definition, emerging data suggest that malignant clones may already exist at the time of eradication and progress rapidly [3]. Excluding such early-onset cases may inadvertently remove a distinct biological subset and bias the cohort toward indolent disease. Future work should analyze the entire temporal spectrum or at least report the number and features of excluded early cases.

Fourth, the propensity-score matching balanced only baseline demographics and endoscopy counts, but not proton-pump inhibitor (PPI) indication, dosage, or duration before eradication. PPI use >1 year was markedly higher in the eradicated group (74% vs 24%), yet GERD severity, Helicobacter status prior to eradication, and concurrent medications were not adjusted for. Residual confounding by indication could therefore exaggerate the apparent association between long-term PPI therapy and post-eradication carcinogenesis.

Fifth, the inter-observer agreement for endoscopic atrophy, map-like redness and histological grading was not reported. The Kimura-Takemoto classification and OLGA/OLGIM staging are known to exhibit moderate κ values (0.4–0.6) even among expert endoscopists [4]. Absence of a central review panel raises concern for misclassification bias, especially for subtle lesions in a post-eradication mucosa that often lacks typical *H. pylori*-related inflammation.

Finally, the sample size of the eradicated group (*n* = 81 before matching, *n* = 62 after) limits the precision of multivariable analyses. The 95% confidence interval for ‘mucosal map-like redness’ (OR 9.26–111.77) is extremely wide, and the study had only 80% power to detect an OR ≥3.5 for variables with prevalence <20%. External validation in larger, multicenter cohorts—preferably across regions with varying GC incidence—is warranted before these characteristics are adopted into surveillance guidelines.

In summary, Liu et al. have provided an important descriptive framework for post-eradication EGC. We hope that addressing the above limitations—through prospective design, rigorous eradication confirmation, inclusion of early-onset cases, adjustment for PPI confounders, central pathology review, and expanded sample size—will strengthen the evidence base and refine risk-stratified surveillance strategies.

## Data Availability

Data sharing is not applicable to this article as no new data were created or analyzed in this study.
